# Diagnosis based image quality assessment and enhancement for low dose CT image

**DOI:** 10.3389/fradi.2025.1704113

**Published:** 2025-12-17

**Authors:** B. Nirupama, B.S. Dhevdharsan, U. Shreya Reddy, J. Joshan Athanesious, S. Kiruthika

**Affiliations:** School of Computer Science, Vellore Institute of Technology, Chennai, India

**Keywords:** automated quality assessment, feature extraction, image enhancement, Laplacian sharpening, low-dose CT, medical image analysis, no-reference image quality assessment (NRIQA), quality score prediction

## Abstract

Low-dose Computed Tomography (CT) imaging minimizes radiation exposure but often results in degraded image quality, making diagnosis challenging. Image Quality Assessment (IQA) is a process of quantitatively evaluating the visual quality of images and plays a crucial role in determining whether these CT scans meet the necessary standards for accurate diagnosis. IQA methods help identify issues such as noise, blurriness, or artifacts that may compromise the diagnostic value of the scans. Traditional quality assessment measures how closely an image matches an ideal or reference image. Since obtaining a high-quality reference image is often challenging, an automated quality assessment framework (diagnosis based IQA) using No-Reference Image Quality Assessment (NRIQA) techniques is proposed, allowing quality evaluation and eliminating the need for a high-quality reference image. In this approach, various statistical and structural features are extracted from low-dose CT scans and mapped to radiologist-assigned quality scores, which are subjective evaluations given by experts to train and compare various predictive models. The framework undergoes 100-fold validation, to ensure the reliability of the proposed model. CT images with predicted quality scores of 2 and below undergo spatial domain enhancement to improve their diagnostic value. These enhanced images are then reassessed using the diagnosis based IQA (trained Support Vector Regression) model, demonstrating an improvement in predicted quality scores. In addition, the enhanced images were verified by a radiologist, confirming the effectiveness of the enhancement process. This two-stage approach, automated NRIQA-based quality prediction and selective enhancement provides a reliable, and objective method for assessing and improving low-dose CT image quality.

## 1 Introduction

Computed Tomography (CT) imaging is a critical tool in modern medicine, offering high-resolution, cross-sectional images that aid in disease diagnosis and monitoring. However, radiation exposure associated with conventional high-dose CT (HDCT) scans has raised concerns, particularly for patients requiring frequent imaging. To address this, Low-Dose CT (LDCT) imaging has gained prominence, utilizing reduced radiation doses to minimize health risks. While LDCT significantly lowers radiation exposure, at times it comes at the cost of reduced image quality, manifesting as increased noise and artifacts. These limitations can affect the accuracy of diagnoses from LDCT images, making it essential to develop methods to objectively evaluate and improve their quality, ensuring they remain useful for medical diagnosis. To ensure the diagnostic reliability of LDCT images, an objective image quality assessment (IQA) system is required. Traditionally, radiologists assign quality scores based on subjective evaluation, but this method is human-dependent, prone to bias and lacks standardization. As a result, there is a growing need for automated and objective assessment methods. This has led to the development of various IQA techniques, many of which rely on reference-based methods that compare an image against a high-quality reference. However, in practical medical imaging scenarios, a perfect reference is often unavailable, making such approaches unsuitable for LDCT evaluation. To overcome this limitation, this research employs a No-Reference Image Quality Assessment (NRIQA) approach, in which image quality is assessed without needing a pristine reference. NRIQA plays a crucial role in medical image quality assessment, particularly in modalities like Magnetic Resonance Imaging (MRI), CT, and ultrasonic imaging. This method provides an objective evaluation of image quality, which is critical for ensuring that images are of sufficient quality for accurate diagnosis and treatment planning. NRIQA approach adapts well to the variability in imaging conditions, such as differences in patient sizes, movement artifacts, or imaging protocols, by assessing image quality based on inherent features. With the increasing use of advanced imaging techniques and machine learning algorithms in medical imaging, NRIQA is vital for evaluating their performance without relying on traditional reference-based metrics. Ultimately, high-quality images are essential for clinical decision-making, and NRIQA helps ensure that images used in clinical settings are evaluated consistently, thereby supporting better patient care. This study proposes a diagnosis based NRIQA model for LDCT imaging, leveraging statistical and structural features to predict quality scores without the need for a high-quality reference image. This model integrates feature selection, machine learning, and targeted image enhancement, providing an automated, consistent, and reliable solution for LDCT quality assessment, reducing human dependency and enhancing diagnostic reliability. The rest of the article is organised as follows. Section 2 describes the literature survey and the design of proposed model is discussed in Section 3. Experimental results are discussed in the Section 4, followed by Section 5, discussed the performance of proposed model in clinical evaluation. Finally, in Section 6 conclusion and future work is discussed.

## 2 Related works

No-reference image quality assessment (NR-IQA) has been widely explored in medical and non-medical imaging. Several studies focus on NR-IQA for MRI and CT scans, deep learning-based assessments, feature extraction, and medical image enhancement. Enhancement techniques improve contrast, sharpness, and noise reduction, complementing NR-IQA for better diagnostics.

The research by Wang et al. (1) and Stepień and Oszust (2) developed NR-IQA methods for MRI scans by incorporating automated distortion recognition and deep learning-based fusion of multiple architectures, respectively. The study by Wang et al. (1) employed grayscale normalization, gamma correction, and Gaussian filtering, followed by feature extraction using MSCN coefficients and AGGD analysis, achieving a 79.6x improvement in Quality Index (QI) over Contrast-to-Noise Ratio (CNR). Meanwhile, Stepień and Oszust (2) utilized ResNet18 and ResNet50 for feature extraction and applied PCA and Support Vector Regression (SVR), outperforming state-of-the-art techniques with high correlation to radiologists’ assessments. Similarly, the work by Shen et al. (3) introduced an NR-IQA method for low-dose CT (LDCT) images using sparse representation and a Just Noticeable Distortion (JND) model. This method combined JND maps and sparse features into a regression model for quality prediction, demonstrating high correlation with radiologists’ subjective scores.

Deep learning techniques have played a significant role in advancing NR-IQA. The research by Golestaneh et al. (4) introduced TReS, a hybrid NR-IQA model integrating Convolutional Neural Networks (CNNs) and Transformers to extract both local and non-local features. Their approach improved accuracy using relative ranking and self-consistency mechanisms. Likewise, Yang et al. (5) presented MANIQA, which leveraged Vision Transformers (ViT) with attention mechanisms to enhance GAN-based distortion assessment. This method outperformed existing techniques across four datasets and won the NTIRE 2022 Perceptual Image Quality Assessment Challenge. The work by Smith et al. (6) proposed an NR-IQA guided cut-off point selection strategy to refine dataset selection for fine-grained classification, improving model accuracy by filtering out low-quality images. Additionally, the research by Varga et al. (7) proposed a decision fusion approach using multiple pre-trained CNNs, such as VGG16, ResNet50, and InceptionV3, to enhance perceptual image quality estimation.

Efforts to reduce computational complexity in NR-IQA have been explored by Huang and Fang (8), which introduced a lightweight parallel framework (LPF) utilizing a pre-trained VGG16 network, a Feature Embedding Network (FEN), and a Distortion-Aware Quality Regression Network (DaQRN). The model’s parallel training improved convergence speed while maintaining superior performance compared to traditional BIQA methods. Similarly, the approach by Bagade et al. (9) proposed a machine learning-based NR-IQA metric for JPEG images, extracting Image Gradient (IG), Spatial Frequency Measure (SFM), and Spectral Activity Measure (SAM) features, and employing Artificial Neural Networks (ANN) and Neuro Fuzzy Classifiers (NFC). ANN demonstrated higher accuracy than NFC, highlighting its computational efficiency.

The research by Shi et al. (10) proposed BMEFIQA, a blind quality assessment method for multi-exposure fused (MEF) images, evaluating structural integrity, naturalness, and colorfulness using gradient similarity, exposure weighting, and entropy-based features. Random Forest regression was used for quality prediction, outperforming several existing methods on public MEF databases.

Several studies have integrated feature extraction techniques with classification models for disease detection in medical imaging. The study by Chowdhary and Acharjya (11) reviewed various segmentation and feature extraction techniques for medical images, highlighting the effectiveness of clustering-based segmentation and Haralick texture features. Sharma et al. (12) developed an automated bone cancer detection system using Histogram of Oriented Gradients (HOG) and Gray-Level Co-occurrence Matrix (GLCM) features, achieving an F1-score of 92.68% with Support Vector Machine (SVM). Likewise, (13) combined handcrafted Local Binary Pattern (LBP) and deep learning features from pre-trained CNNs to detect COVID-19 in CT scans. Their hybrid approach using VGG19+LBP achieved a classification accuracy of 99.4%, demonstrating the effectiveness of feature fusion.

COVID-19 detection using machine learning was explored by Imani (14), where contextual features were extracted using morphological filters, Gabor filter banks, and attribute filters. A shallow CNN was used for feature reduction, with SVM and Random Forest achieving 94% accuracy for X-ray images and 76% for CT scans. In lung cancer detection, the work by Alzubaidi et al. (15) compared global and local feature extraction approaches using CT scans. The best results were achieved with local feature extraction, where SVM combined with Gabor Filter features obtained 97% accuracy, 96% sensitivity, and 97% specificity, demonstrating the superiority of localized learning.

The research by Salem et al. (16) explores various histogram-based contrast enhancement techniques for medical images. The researchers implemented the methods using MATLAB and evaluated their performance on different medical images based on Peak Signal-to-Noise Ratio (PSNR), Mean Square Error (MSE), and Standard Deviation (SD). Their results demonstrated that Quadratic Dynamic Histogram Equalization and Contrast-Limited Adaptive Histogram Equalization were the most effective methods.

The sharpening enhancement technique for MR images proposed by Jeevakala (17) uses Laplacian Pyramid (LP) and Singular Value Decomposition (SVD) to improve edge visibility and segmentation. The LP decomposition preserves shape and texture information, while SVD enhances contrast at object boundaries. The method applies one level of LP decomposition to extract edge details, followed by SVD-based contrast enhancement.

The reviewed studies highlight advancements in NR-IQA, deep learning-based assessments, feature extraction, and enhancement techniques. Hybrid CNN-Transformer models, lightweight frameworks, and contrast enhancement methods have significantly improved image quality assessment and disease detection. These findings support developing an NR-IQA approach for LDCT scans by integrating feature extraction, quality estimation, and enhancement for optimal diagnostics.

## 3 Methodology

**Figure 1:**
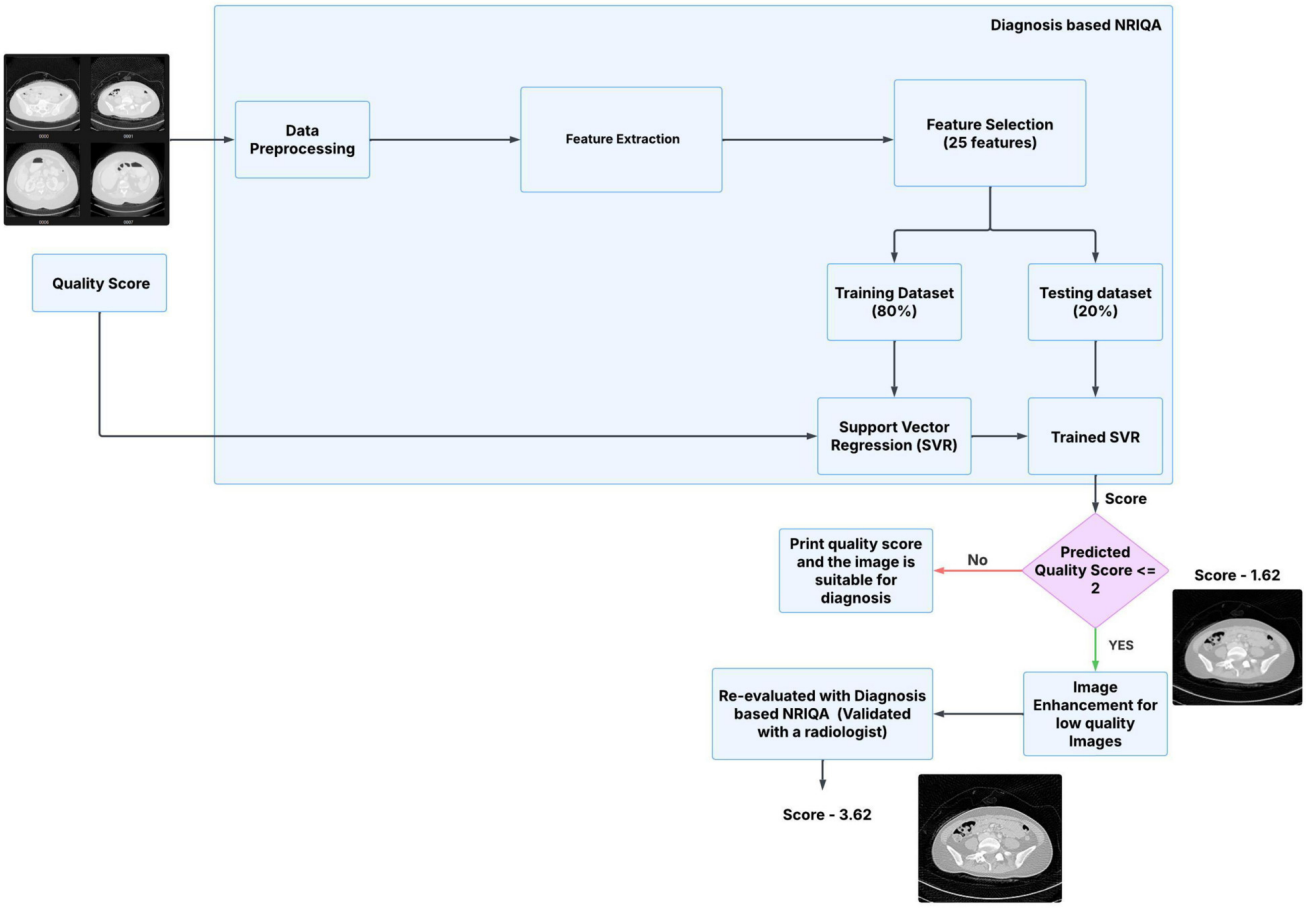
Architecture of diagnosis based image quality assessment and enhancement model.

The process of assessing and enhancing the quality of LDCT images plays a vital role in ensuring accurate diagnostics while maintaining reduced radiation exposure. This study presents an integrated framework that combines NRIQA and targeted enhancement for LDCT imaging. Utilizing a robust feature extraction mechanism, including texture, edge, and frequency-based metrics, the framework employs SVR to predict quality scores and identify low-quality images. To address diagnostic usability, Laplacian sharpening is applied to images with low predicted quality scores, verified through collaboration with radiologists. Figure 1 shows the workflow of the methodology. This approach not only bridges the gap between quality assessment and enhancement but also aims to improve the reliability and applicability of LDCT imaging in clinical practice.

### 3.1 Dataset description

**Figure 2:**
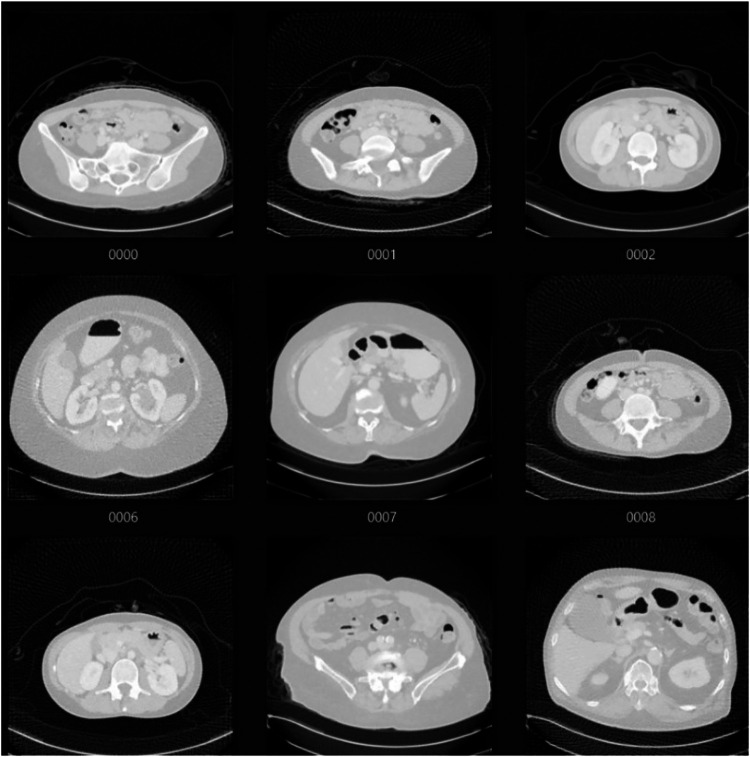
Sample scan images from the dataset without preprocessing.

The dataset used in this study consists of 1,000 LDCT scan images, obtained from Lee et al. (18), each assigned a continuous quality score from 0 to 4 by experienced radiologists. Sourced from the LDCTIQAC2023 repository, the dataset contains real-world annotations, making it highly relevant for clinical applications. The images, stored in .tif format, were acquired using an abdominal soft-tissue window (width/level: 350/40) to ensure consistency. Figure 2 illustrates a sample of the raw dataset used for this study. To introduce variability, the dataset includes four noise levels, four artifact levels, and 16 combinations of noise and artifacts, generated by adjusting noise levels, projection stack size, and angular increments. The quality assessment was performed by five radiologists, each with over 10 years of experience, from Seoul Metropolitan Government Seoul National University Boramae Medical Center and Veterans Health Service Medical Center in South Korea. A five-point Likert scale was used to evaluate image noise, anatomical structure clarity, and diagnostic interpretability. The final subjective quality score was obtained by averaging the individual ratings to minimize bias.

**Figure 3:**
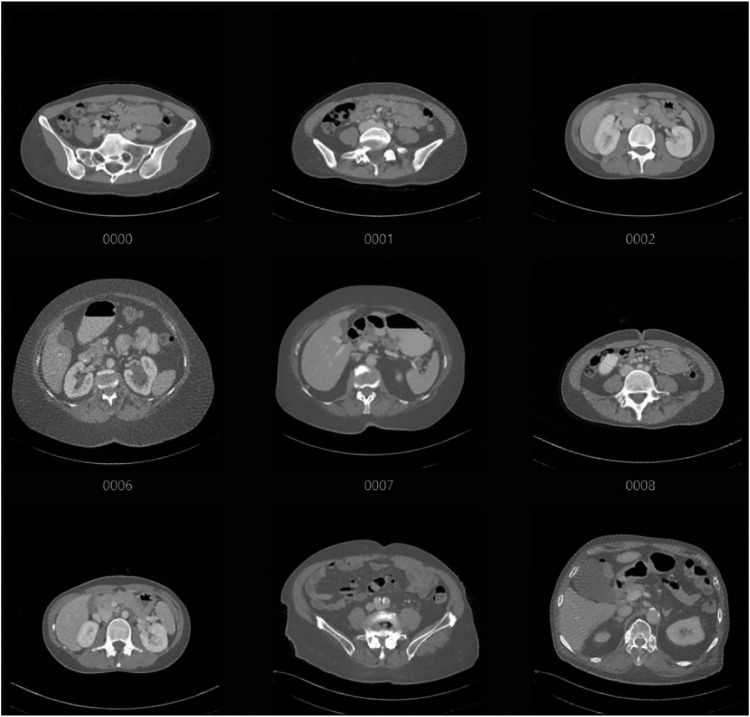
Sample images after preprocessing shown (images shown in Figure 2.

This dataset provides a robust foundation for training models to assess image quality and predict scores for unseen CT scans. Table 1 represents the scoring criteria for the CT images present in the dataset. In order to prepare the dataset for analysis, a series of pre-processing steps are performed to ensure the images are in a suitable format for machine learning model training. All images are resized to a standard size of 224 × 224 pixels to maintain consistency and ensure compatibility with the chosen models. The images were then normalized to a range of [0,1], ensuring uniform intensity values across the entire dataset. Additionally, each image was converted to an 8-bit format to optimize processing. Finally, after processing, the images are converted back into an appropriate format for further analysis, typically a PIL image format, allowing for seamless integration with Python-based libraries. Figure 3 depicts a sample of the dataset after pre-processing.

**Table 1:** Image quality scoring criteria for computed tomography data.

Score	Quality	Description
0	Bad quality	Desired features are not shown
1	Poor quality	Diagnostic interpretation is impossible
2	Fair quality	Suitable for limited clinical interpretation
3	Good quality	Suitable for diagnostic interpretation
4	High quality	The anatomical structure is evident

### 3.2 Feature extraction

To effectively capture the various characteristics of the CT scans, a set of features is extracted from each image. These features include intensity-based metrics, histogram based metrics, texture-based metrics using the Gray-Level Co-Occurrence Matrix (GLCM) and Local Binary Pattern (LBP), frequency-based features obtained through fast fourier transform, edge-based and shape-based features.

The intensity based features, namely min, max, mean, median, standard deviation, range, skewness, kurtosis, 10th, and 90th percentile values are extracted from the image. From the distribution of the pixel values, the entropy, energy, and mode are calculated. By generating the edge map from the image, the mean, variance, edge count, area, perimeter, circularity, and compactness values are generated. The sharpness score, tissue density, texture heterogeneity, signal to noise, and contrast to noise ratio values are calculated because they play an major role in visualisation. From the frequency domain of an image the sum, max, mean, power spectrum flatness, and sum of frequencies higher than the mean are calculated. Finally, to analyze the texture properties of the CT image, the features are extraced from gray level co-occurrence matrix (with the distance of 1 and the angle of 0 degree) and local binary pattern (for the radius of 1 and circular neighborhood of 8 points). The mean and variance are computed from the local binary pattern of an image. The contrast, correlation, energy, homogeneity, dissimilarity, entropy, and max probability are computed using gray level co-occurrence matrix. After extracting these features, they are mapped to the corresponding quality scores (provided in the dataset) and stored in a CSV file for further analysis.

### 3.3 Model training and comparative study

Several machine learning models are evaluated to determine the most effective approach for predicting the quality scores of CT images. The models tested include Random Forest, Multi-Layer Perceptron (MLP) Regressor, XGBoost, and SVR. The dataset is split into 80% training and 20% testing to ensure reliable performance evaluation. Further, each model is trained using the extracted features to learn the relationship between image characteristics and quality scores. Hyperparameter tuning is performed using Random Search and Grid Search for the SVR model to fine-tune model parameters. The predicted quality scores are compared against the ground truth values using error metrics and correlation coefficients. Ultimately, SVR tuned with Grid Search yields the best results.

### 3.4 Feature selection and validation

**Figure 4:**
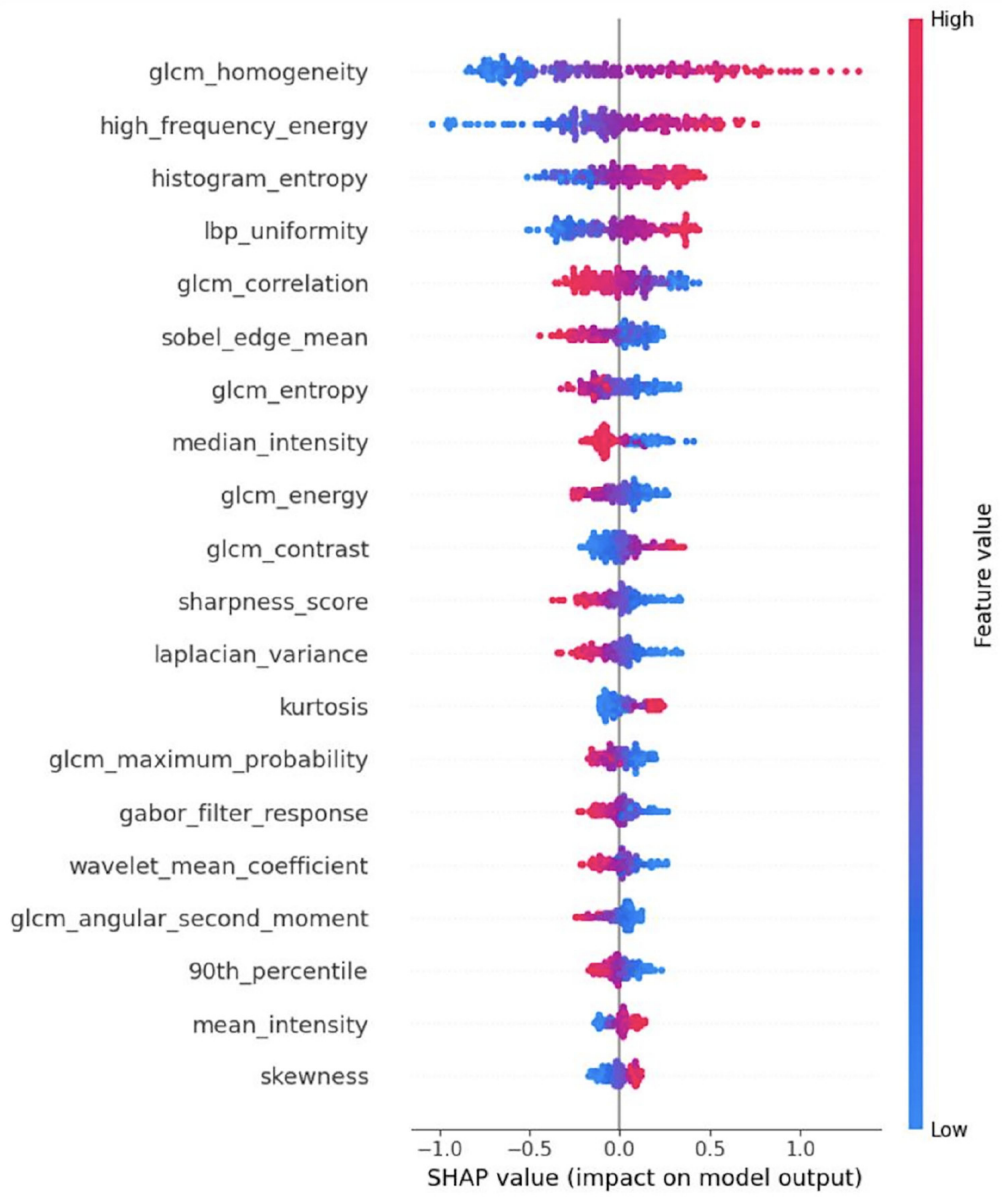
SHAP analysis on the extracted features.

**Figure 5:**
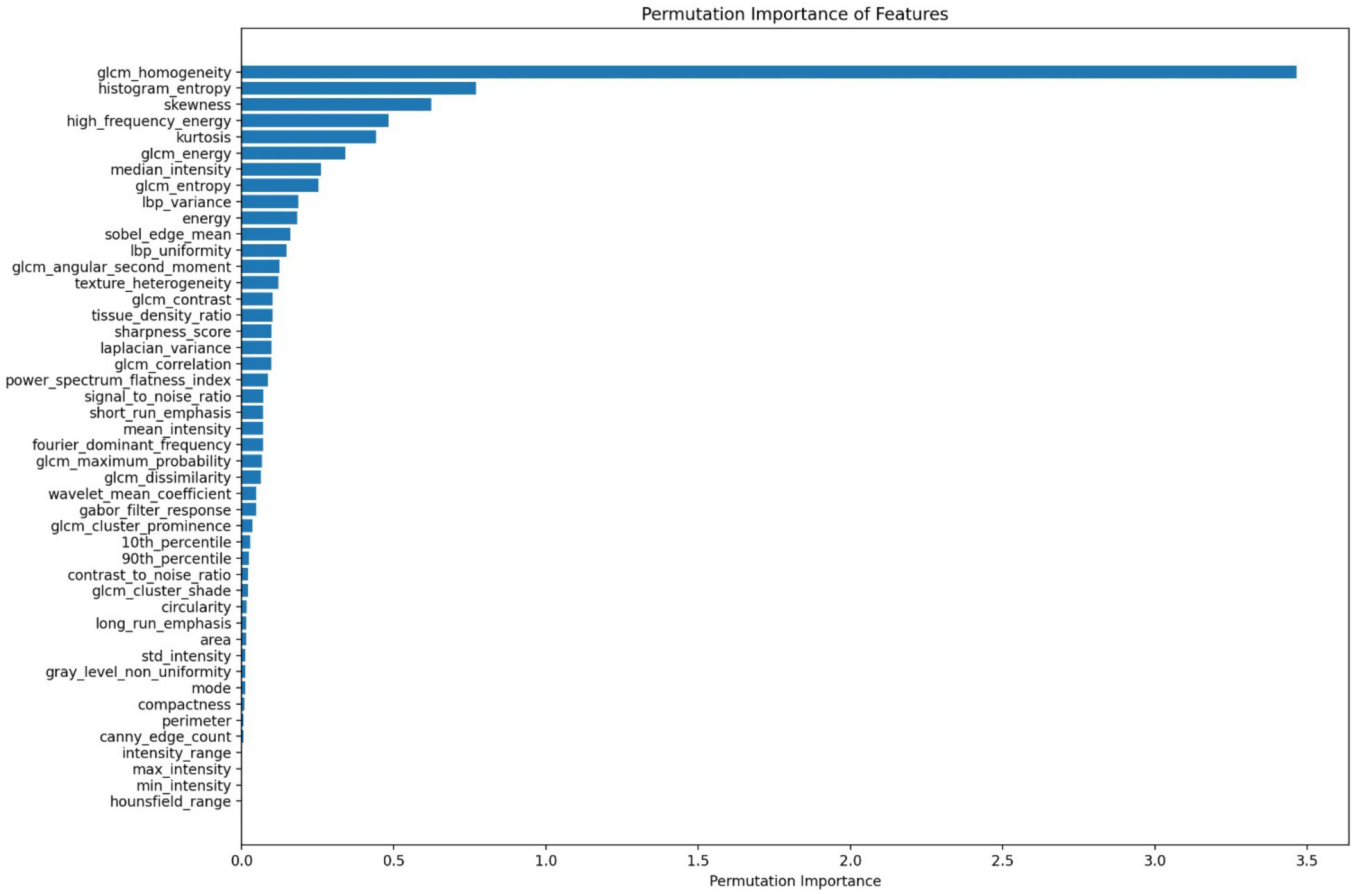
Permutation feature importance to select top 25 features.

To improve the model’s performance, feature selection is performed using SHAP analysis as shown in Figure 4 and permutation importance as shown in Figure 5. From the extracted features, the 25 most relevant ones are selected, and the SVR model is then retrained using this optimized feature subset. A 100-fold cross-validation approach is employed on SVR with Grid Search (SVR-GS) to evaluate the model’s performance, ensuring model robustness and minimizing overfitting.

### 3.5 Image enhancement for low-quality images

**Figure 6:**
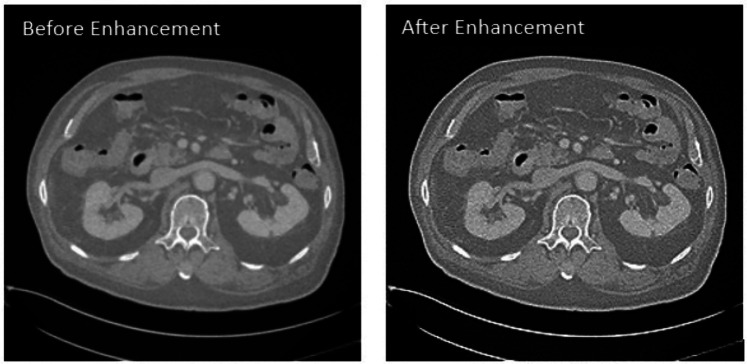
Images before and after applying Laplacian Sharpening for enhancement.

This study applies a Laplacian-based sharpening technique to enhance low-quality abdominal CT images, specifically those with predicted quality scores of 2 and below. Laplacian sharpening is implemented by applying the Laplacian operator to detect edges and enhance them by subtracting a fraction of the Laplacian response from the original image. The enhanced images as depicted in Figure 6 are validated by the radiologist and re-evaluated using the trained SVR model, confirming improved quality scores.

### 3.6 Experimental set-up

The entire methodology is implemented using Python and several key libraries, including Scikit-learn, OpenCV, and PIL. Model training and evaluation are conducted on high-performance computing resources with GPU acceleration. Google Cloud Platform (GCP) was utilized with a T4 GPU core for training, providing robust computational power for handling extensive image processing tasks. This implementation ensures scalability and efficiency for future applications. This methodology provides a systematic, data-driven approach to evaluating and improving the quality of LDCT images, minimizing human dependence on traditional quality assessment methods and enhancing diagnostic reliability.

## 4 Results and discussion

Several machine learning algorithms, Random Forest, XGBoost, MLP Regressor, SVR with Random Search (SVR-RS), SVR-GS, and SVR with 100-fold cross-validation (SVR - 100CV) are evaluated on various error and correlation metrics. Table 2 depicts the comparative study between various models. Among these, SVR-GS achieves the best results, demonstrating superior accuracy in predicting image quality. This model showed lower error values and higher correlation scores, making it more reliable for assessing image quality.

**Table 2:** Error metrics.

Model	Mean squared error	*R* Squared	Mean absolute error	RMSE	Explained variance	Median absolute error	PLCC	SROCC	KLCC
Random forest	0.0998	0.9084	0.2327	0.3159	0.9084	0.1669	0.9532	0.9461	0.8289
XGBoost	0.0952	0.9126	0.2292	0.3086	0.9126	0.1664	0.9553	0.9403	0.8338
MLP regressor	0.0805	0.9262	0.2254	0.2837	0.9263	0.1893	0.9635	0.9609	0.8512
SVR-RS	0.0717	0.9343	0.2143	0.2677	0.9343	0.1911	0.9667	0.9651	0.8600
SVR-GS	0.0673	0.9383	0.2081	0.2594	0.9383	0.1679	0.9690	0.9678	0.8662
SVR-100CV	0.0651	0.9221	0.1996	0.2475	0.9284	0.1692	0.9671	0.9394	0.8671

### 4.1 Evaluation of correlation metrics

Pearson Linear Correlation Coefficient (PLCC) measures the linear relationship between predicted and actual scores. A PLCC value close to 1 indicates a strong linear correlation. It is computed as:

$$\text{PLCC}=\frac{\sum (V_{\;p,i}-{\bar{V}}_{p})(V_{a,i}-{\bar{V}}_{a})}{\sqrt{\sum (V_{\;p,i}-{\bar{V}}_{p}{)}^{2}\sum (V_{a,i}-{\bar{V}}_{a}{)}^{2}}}$$

where $V_{\;p,i}$ and $V_{a,i}$ are the predicted and actual scores, and ${\bar{V}}_{p}$ and ${\bar{V}}_{a}$ are their respective means. For the SVR-100CV model, the PLCC score is 0.9671.

Spearman Rank-Order Correlation Coefficient (SROCC) is a non-parametric measure that evaluates the monotonic relationship between predicted and actual scores. A SROCC value close to 1 indicates a stronger ordinal relationship. It is given by:

$$\text{SROCC}=1-\frac{6\sum R_{i}^{2}}{S(S^{2}-1)}$$

where $R_{i}$ is the rank difference between corresponding pairs, and S is the total number of samples. For the SVR-100CV model, the SROCC score is 0.9394.

Kendall’s Tau-b (KROCC) assesses the strength and direction of association between two ranked variables while adjusting for ties. A KROCC value closer to 1 signifies better agreement between rankings. It is defined as:

$$\tau_{b}=\frac{S_{c}-S_{d}}{\sqrt{\left(S_{c}+S_{d}+T_{p})(S_{c}+S_{d}+T_{a}\right)}}$$

where $S_{c}$ and $S_{d}$ represent the number of concordant and discordant pairs, respectively, while $T_{p}$ and $T_{a}$ account for tied ranks in each variable. For the SVR-100CV model, the KROCC score is 0.8671.

**Figure 7:**
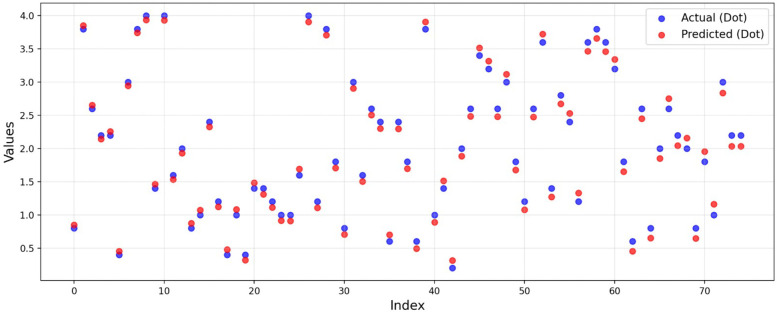
Actual (plotted as blue circle) and Predicted (plotted as red circle) quality scores for the sample test images.

These correlation metrics provide a robust assessment of the model performance by evaluating both linear and rank-based relationships between predicted and actual quality scores. The similarity between the actual and predicted scores can be seen in Figure 7. This figure represents the scores of 25% of the test images for better visualization.

### 4.2 Evaluation of error metrics

To assess the predictive accuracy of the models, we evaluated six error-based metrics:

Mean Squared Error (MSE) calculates the average squared difference between actual values ($V_{\text{actual}}$) and predicted values ($V_{\text{\textbackslash{},predicted}}$). A lower MSE indicates better model performance. N represents the total number of samples, while $V_{\text{actual},i}$ and $V_{\text{\textbackslash{},predicted},i}$ denote the actual and predicted values for the ith sample. For the SVR-100CV, MSE value is 0.0651.

$$\text{MSE}=\frac{1}{N}\sum_{i=1}^{N}(V_{\text{actual},i}-V_{\text{\textbackslash{},predicted},i}{)}^{2}$$

*R*-Squared ($R^{2}$) represents the proportion of variance in the dependent variable ($V_{\text{actual}}$) that is predictable from the independent variables. A higher $R^{2}$ value (closer to 1) indicates a better model fit. $V_{\text{mean}}$ represents the mean of actual values, and the summations compute the total variance explained by the model. For the SVR-100CV, $R^{2}$ value is 0.9221.

$$R^{2}=1-\frac{\sum (V_{\text{actual},i}-V_{\text{\textbackslash{},predicted},i}{)}^{2}}{\sum (V_{\text{actual},i}-V_{\text{mean}}{)}^{2}}$$

Mean Absolute Error (MAE) calculates the average absolute difference between actual values ($V_{\text{actual}}$) and predicted values ($V_{\text{\textbackslash{},predicted}}$), making it less sensitive to outliers than MSE. N is the total number of samples, and absolute differences are computed for each prediction. For SVR-100CV, MAE value is 0.1996.

$$\text{MAE}=\frac{1}{N}\sum_{i=1}^{N}|V_{\text{actual},i}-V_{\text{\textbackslash{},predicted},i}|$$

Root Mean Squared Error (RMSE) measures the standard deviation of residuals (prediction errors), providing a more interpretable error metric than MSE. N is the number of samples, and residuals represent the differences between actual and predicted values. For SVR-100CV, RMSE value is 0.2475

$$\text{RMSE}=\sqrt{\frac{1}{N}\sum_{i=1}^{N}(V_{\text{actual},i}-V_{\text{\textbackslash{},predicted},i}{)}^{2}}.$$

Explained Variance Score (EVS) evaluates how well a model captures variance in the target variable ($V_{\text{actual}}$), with a value closer to 1 indicating a better model. $\text{Var}(V_{\text{actual}})$ represents the variance of actual values, while $\text{Var}(V_{\text{actual}}-V_{\text{\textbackslash{},predicted}})$ measures the variance of residuals. For SVR-100CV, EVS value is 0.9284

$$\text{EVS}=1-\frac{\text{Var}(V_{\text{actual}}-V_{\text{\textbackslash{},predicted}})}{\text{Var}(V_{\text{actual}})}.$$

Median Absolute Error (MedAE) computes the median of absolute differences between actual ($V_{\text{actual}}$) and predicted values ($V_{\text{\textbackslash{},predicted}}$), offering robustness to outliers. The median operation is applied to the absolute differences between actual and predicted values. For SVR-100CV, MedAE value is 0.1692.

$$\text{MedAE}=\text{median}(|V_{\text{actual},i}-V_{\text{\textbackslash{},predicted},i}|)$$

Among the evaluated models, SVR-GS achieved the best results across all error metrics, as depicted in Table 2. However, to mitigate overfitting and ensure generalizability when comparing results with algorithms from other studies, SVR-100CV scores are employed as the primary benchmark for comparison.

Table 3 presents a comparative analysis of various models used for quality assessment, highlighting the superior performance of the proposed SVR-100 CV model. The Just Noticeable Difference (JND)-based models, from Shen et al. (3) including (19–23), use sparse representation to predict image quality and follow an 80-20 data split. These models achieve high correlation metrics, with PLCC values ranging from 0.923 to 0.955, SROCC between 0.919 and 0.953, and KLCC between 0.771 and 0.827. The No-Reference Image Quality Assessment (NRIQA) algorithms, such as SNR, CHO, NPWE, BRISQUE, and NIQE, operate with a 70-30 data split exhibit lower correlation values, with PLCC scores between −0.7278 and 0.8226.

**Table 3:** Comparison of models.

Model name	PLCC	SROCC	KLCC
**SVR-100 CV**	**0.9671**	**0.9394**	**0.8671**
Chou JND (3, 19)	0.955	0.953	0.823
Yang JND (3, 23)	0.954	0.952	0.827
Liu JND (3, 20)	0.951	0.949	0.822
Wu JND (3, 22)	0.931	0.924	0.780
Wan JND (3, 21)	0.923	0.920	0.771
Zhang (3, 24)	0.925	0.919	0.775
SNR (18)	0.8226	0.8748	0.7052
CHO (18)	0.3778	0.4395	0.3159
NPWE (18, 25)	0.3566	0.4165	0.2982
BRISQUE (18, 26)	0.7500	0.7863	0.5856
NIQE (18, 27)	−0.7278	−0.7143	−0.5339

Bold value indicates the scores of proposed model.

In contrast, the proposed SVR-100 CV model, which utilizes feature-based extraction and follows an 80-20 data split, outperforms both the mentioned JND models as well as the approaches proposed in (18), achieving the highest correlation metrics in PLCC at 0.9671 and KLCC at 0.8671. This demonstrates the effectiveness of the feature-based extraction approach in accurately predicting image quality scores.

### 4.3 Evaluation of enhancement

**Figure 8:**
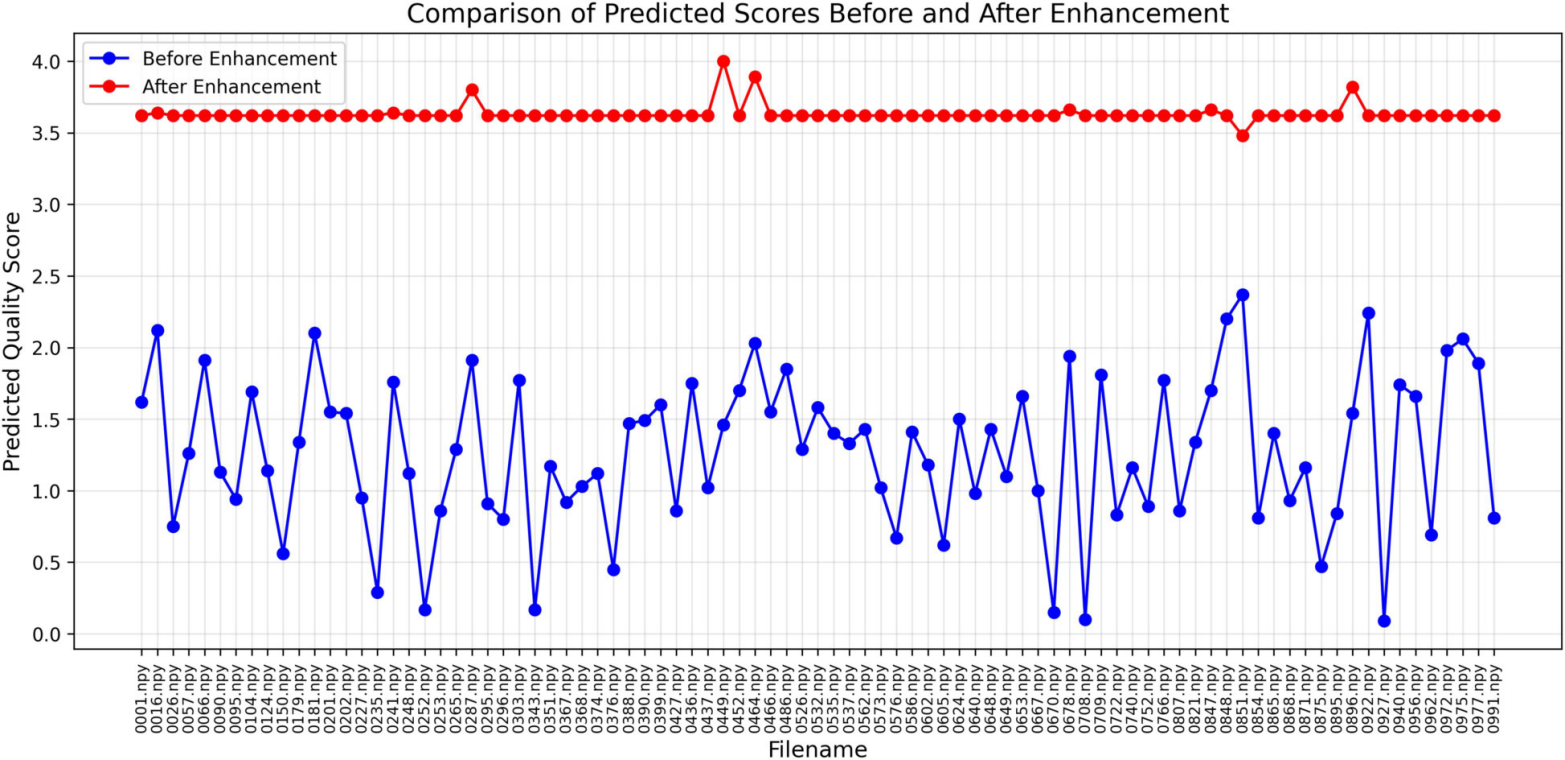
Predicted scores before (plotted as blue circle) and after enhancement (plotted as red circle).

The effectiveness of Laplacian sharpening was evaluated by re-testing the enhanced images using the trained SVR-GS model. The results showed an improvement in predicted quality scores as shown in Figure 8, indicating a measurable enhancement in image quality. The graph demonstrates a significant improvement in predicted quality scores for abdominal CT images after enhancement, as indicated by the consistent higher values in red compared to the fluctuating lower scores before enhancement in blue. By incorporating objective re-evaluation and expert validation, this enhancement approach not only improves perceptual image quality but also ensures that low-qualit LDCT images become more reliable for clinical interpretation.

## 5 Clinical evaluation

To ensure an unbiased assessment, the dataset was randomly shuffled before being presented to radiologist Dr. Inthulan Thiraviaraj, a Consultant and Head at Athipathi Scan Centre with extensive experience in neuroradiology, diagnostic imaging, and fetal medicine. The dataset consisted of three categories: unenhanced low-quality images(score less than 2), the same low-quality images enhanced using Laplacian sharpening, and unenhanced high-quality images. Without prior knowledge of each image’s category, the radiologist evaluated 130 images on a screen with a native display resolution of 2,880 by 1864. Dr. Inthulan assigned scores ranging from 0 to 4 based on visual clarity and diagnostic usability. This blinded evaluation method ensured that any observed improvements in image quality were solely attributed to the enhancement process rather than preconceived biases.

During the assessment, the radiologist carefully examined anatomical structures, edge definition, and noise levels to determine the diagnostic usability of each image. Among the enhanced images, 96% were correctly identified as enhanced, demonstrating a clear visual distinction from the unenhanced low-quality images. Furthermore, 88% of the enhanced images were assigned a high-quality score of 3, confirming that Laplacian sharpening significantly improved image clarity. These findings underscore the effectiveness of the enhancement process in refining the perceptual quality of low-dose CT scans, making them more interpretable for clinical diagnosis.

**Figure 9:**
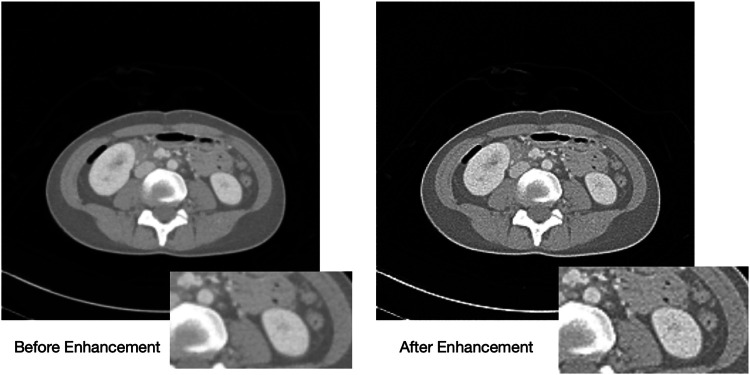
Visualizing the soft tissue before (left) and after (right) enhancement.

**Figure 10:**
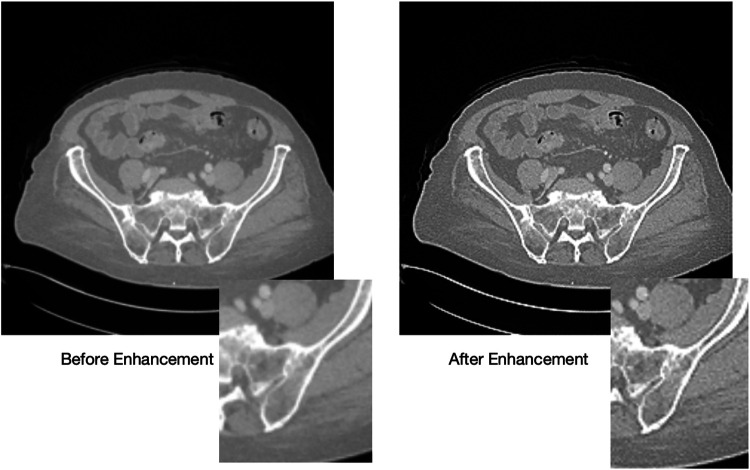
Visualizing the bone tissue before (left) and after (right) enhancement.

The enhanced images exhibit variations in enhancement based on the tissue type. Some images display improvements in the soft tissue CT window as shown in Figure 9, while others show enhancements in the bone tissue window as shown in Figure 10. Soft tissues, including muscles, organs, and fat, require contrast enhancement for better visualization, whereas bone tissues, such as the skeletal structure, benefit from sharper edge definition. The radiologist confirms that the enhancement process effectively improves the clarity of soft tissues in some cases and bone structures in others, depending on the image characteristics.

## 6 Conclusion and future work

This study systematically evaluates multiple models for automated image quality assessment in LDCT imaging. While models such as Random Forest and MLP Regressor demonstrated reasonable performance, they exhibited higher error rates and lower correlation scores compared to SVR. Random Forest tended to overfit due to its reliance on complex decision boundaries, while MLP Regressor showed inconsistent performance across different validation folds. SVR, optimized using Grid Search, was selected due to its superior generalization ability, achieving the lowest error values and highest correlation scores across all metrics.

To further enhance diagnostic reliability, Laplacian sharpening is applied to images with predicted scores of 2 and below. The enhanced images were retested using the trained SVR model, showing improved quality scores, confirming the effectiveness of this enhancement approach. Additionally, radiologist Dr. Inthulan Thiraviaraj verified the enhanced images, further reinforcing their improved diagnostic value. Despite the promising results, a small percentage of enhanced images were not correctly identified as enhanced, highlighting the need for further refinement in the enhancement process.

This suggests that while Laplacian sharpening significantly improves image clarity, there may be cases where the enhancement is insufficiently pronounced or indistinguishable from unenhanced images. Future work will focus on addressing these limitations by exploring advanced enhancement techniques and optimization strategies to ensure consistent and universally recognizable improvements in image quality, thereby further enhancing the diagnostic utility of low-dose CT scans.

## Data Availability

Publicly available datasets were analyzed in this study. This data can be found here: https://ldctiqac2023.grand-challenge.org/.
